# Chronic Right Hypochondrial Pain Due to a Painful Abdominal Wall Lipoma Mimicking Hepatobiliary Disease: A Case Report

**DOI:** 10.7759/cureus.101673

**Published:** 2026-01-16

**Authors:** Muhammad Ikram, Nazir Shah

**Affiliations:** 1 General Surgery, Hayatabad Medical Complex, Peshawar, PAK; 2 Internal Medicine, Hayatabad Medical Complex, Peshawar, PAK

**Keywords:** abdominal wall pain, anterior cutaneous nerve entrapment, lipoma, right hypochondrial pain, ultrasound

## Abstract

Chronic right hypochondrial pain is most often evaluated in the context of hepatobiliary disease. When routine investigations fail to identify an intra-abdominal cause, alternative sources of pain should be considered. We report the case of a patient with long-standing right upper abdominal pain that was exacerbated by movement and local palpation and had been repeatedly assessed for biliary pathology. Initial imaging suggested a possible gallbladder abnormality, which was not reproduced on subsequent examinations. Further focused assessment revealed a localized lesion with sonographic appearances of lipoma within the abdominal wall at the site of maximal tenderness. The size of the lesion was approximately 1 × 1.4 cm. The clinical features and imaging findings were consistent with an abdominal wall source of pain. This report emphasizes the value of thorough physical examination and targeted ultrasonography in patients with persistent right hypochondrial pain to prevent unnecessary investigations and delays in diagnosis.

## Introduction

Chronic right upper quadrant abdominal pain is a frequent clinical presentation, most often investigated for hepatobiliary pathology. The most frequent causes include cholelithiasis, cholecystitis, choledocholithiasis, and irritable bowel syndrome, while fatty liver disease is a less common contributor. Other chronic hepatobiliary disorders, as well as pathology arising from the right kidney or the lower lobe of the right lung, may also account for right hypochondrial pain in rare cases.

Ultrasonography remains the first-line modality for gallbladder and liver diseases [1]. When imaging repeatedly fails to identify a visceral cause, functional or abdominal wall etiologies are frequently overlooked, leading to unnecessary investigations. Abdominal wall lipomas comprise about 16.1% of all abdominal wall masses [2] and are rarely a cause of abdominal pain [3]. Painful abdominal wall lipomas are a rare but recognized cause of localized abdominal wall pain [3]. The most common mechanism of pain is the entrapment of local nerves passing through the lipoma, the so-called anterior cutaneous nerve entrapment syndrome (ACNES) [4].

Abdominal wall lipomas may be visible or palpable on clinical examination, but in rare cases, they produce a diagnostic challenge when they are non-visible, especially in the fatty abdomen. Common modalities used for the initial diagnosis of abdominal wall lipomas are ultrasound, MRI, or CT scan of the abdominal wall [5]. We present a case with chronic right upper quadrant abdominal pain illustrating this diagnostic pitfall.

## Case presentation

A 55-year-old woman presented with a 10-year history of recurrent right hypochondrial pain. The pain was localized, persistent, and intermittently exacerbated by movements and house hold working. There was no association with meals, nausea, vomiting, jaundice, fever, or weight loss. Medical history revealed that 10 years earlier, a 3-4 mm gallstone was seen in an abdominal ultrasound, which was presumed to have passed/dissolved spontaneously. Subsequent serial ultrasonography examinations over several years consistently showed a normal liver, gallbladder, and common bile duct. The latest ultrasound reported patchy, mildly echogenic areas within an otherwise normal-thickness gallbladder wall. These findings may reflect benign wall changes such as cholesterolosis/adenomyomatosis. 

Despite the previous normal imaging, the pain persisted. Notably, the patient reported that the pain increased with movements and lying on the right-side disturbing sleep, was relieved with non-steroidal anti-inflammatory drug (NSAIDs), and had no relation to food intake. Examination of the abdomen revealed normal inspection and superficial palpation. Deep palpation elicited a pinpoint tender area in the right hypochondrium, prompting suspicion of a localized cause for her pain. Notably, the patient expressed frustration that even pressure from the ultrasound probe reproduced her pain, yet repeated imaging failed to identify the cause. Radiation of the pain along the dermatome (T-7/8) simulated it with gall bladder pain, in which case it radiates to the inferior angle of the scapula. There were no red-flag features, and routine laboratory investigations, including liver function tests, serum amylase, and lipase, were within normal limits.

**Figure 1:**
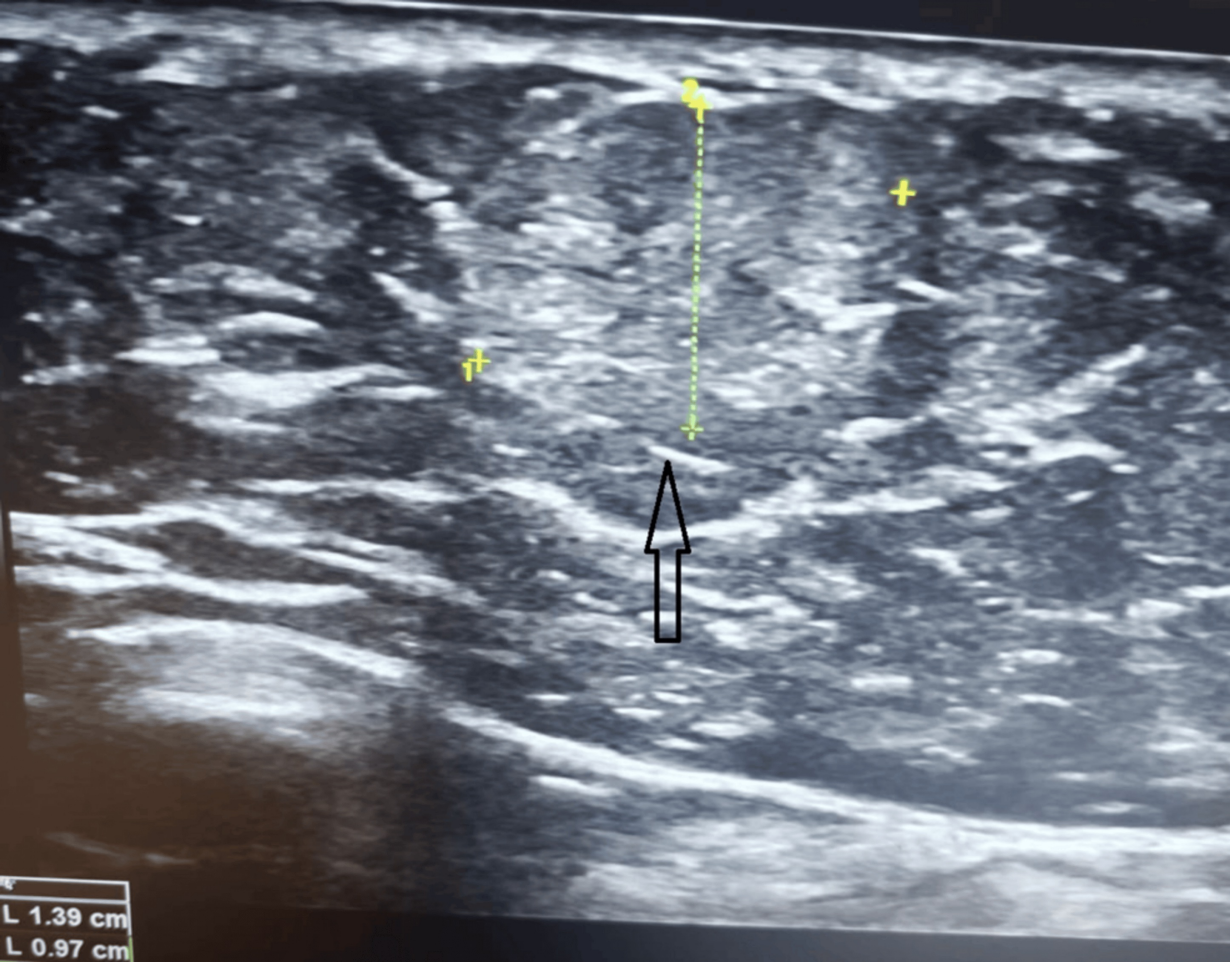
Targeted ultrasound of the right hypochondrium showing a well-defined subcutaneous lipoma (arrow) within the abdominal wall at the site of maximal tenderness.

Initial ultrasound with a standard probe was normal or inconclusive. So, given the localized nature of the pain, a repeat targeted ultrasound examination was performed using an appropriate high-frequency probe placed directly over the point of maximal tenderness. Targeted ultrasonography revealed a well-defined subcutaneous lesion measuring approximately 1 × 1.4 cm within the abdominal wall, consistent with a lipoma, precisely corresponding to the site of maximal pain (Figure 1). In addition, a few other subcutaneous lipomas were identified in adjacent areas; however, these were non-tender. The focal tenderness associated with a single lesion suggests possible entrapment or irritation of a cutaneous nerve with some inflammation. The liver, gallbladder, and bile ducts remained normal. Based on clinical and imaging findings, a diagnosis of abdominal wall pain secondary to a painful subcutaneous lipoma with possible local nerve irritation was made.

Since the patient didn’t consent for local anesthetic trial, and given the localized and pressure-provoked nature of the pain, the patient was managed conservatively with reassurance regarding the benign nature of the condition, avoidance of local pressure, and a therapeutic trial of topical NSAID gel along with capsaicin cream, leading to substantial pain relief and improved functional capacity, allowing the patient to perform household activities without symptom exacerbation. The favorable response further supported an abdominal wall origin rather than visceral pathology. Local long-acting corticosteroid injections (e.g., methylprednisolone acetate or triamcinolone) were considered in the event of inadequate response to topical therapy or if the pain became functionally limiting. Surgical excision was discussed as a definitive treatment option should symptoms persist or worsen.

## Discussion

Abdominal wall pain accounts for a significant proportion of chronic abdominal pain cases but is frequently underdiagnosed [6]. The common causes of concerns, of course, include pathologies of the abdominal viscera like hepatobiliary viscera, peptic ulcer, IBS, urological pathologies, or viscus perforations. Laboratory investigations are commonly normal in such patients [7]. Features suggestive of an abdominal wall origin include localized tenderness, reproducibility with palpation, posture-related worsening, lack of relation to meals, and normal related laboratory investigations, as evident from our case. Misdiagnosis as acute or chronic cholecystitis and peptic ulcer disease is a common problem in such patients [8]. This clinical scenario was similar to that of our patient, in whom the initial diagnosis of a small gall bladder stone was made, which was later presumed to have resolved because of subsequent normal ultrasound examinations, despite persistent symptoms.

Lipomas of the abdominal wall are common benign tumors and are usually asymptomatic [9]. However, when located near sensory nerves or subjected to repeated pressure, they can produce chronic pain mimicking visceral pathology. In this case, the patient underwent repeated hepatobiliary imaging over several years before the true cause was identified. A painful lipoma arising from the abdominal wall or parietal peritoneum may pose an even greater diagnostic challenge because of its deep location, as illustrated in a case report by Bang et al. [9]. Cases have been reported in which patients have been subjected to CT scans of the abdomen or even more costly investigations, suspecting underlying visceral pathologies, which were later proved to be pain related to nerve entrapment [10], lipomas [9], or abdominal wall endometriosis [11].

In this case, a targeted ultrasound examination using a high-frequency probe accurately identified the lesion at the site of maximal tenderness. Previous studies have also demonstrated the utility of ultrasonography in the evaluation of abdominal lipomas, supporting its role as an effective modality for their detection. [12].

Initial management consisted of topical non-steroidal anti-inflammatory drug gel combined with capsaicin cream for analgesia and local anti-inflammatory effect, with consideration of local long-acting corticosteroid injection if the patient’s pain persisted. Although there are no studies specifically evaluating the combination of topical capsaicin and topical NSAID gel for pain due to abdominal wall lipoma, topical capsaicin has been used in similar superficial pain syndromes, such as abdominal wall scar pain and other neuralgic pain conditions [13]. Additionally, systematic reviews and meta-analyses support the efficacy of both topical NSAIDs and capsaicin in localized chronic pain conditions, providing a rationale for their combined use in this case [14].

This case underscores the importance of detailed pain characterization, focused physical examination, and targeted ultrasonography of the abdominal wall. Recognizing abdominal wall causes early can prevent unnecessary advanced imaging, such as CT or MRI, and reduce patient anxiety and healthcare costs.

## Conclusions

Painful subcutaneous lipoma is an uncommon but important cause of chronic right hypochondrial pain. Our patient has a very long history of right hypochondrial pain without any red flag signs, ultimately found to be due to an abdominal wall lipoma. So, we will recommend that in patients with long-standing localized pain and repeatedly normal hepatobiliary imaging, abdominal wall pathology should be actively sought. Targeted ultrasound at the site of maximal tenderness is a simple and effective diagnostic tool.
